# Concentration of fifteen elements in herbaceous stems of *Ephedra intermedia* and influence of its growing soil

**DOI:** 10.1038/s41598-020-72048-w

**Published:** 2020-09-15

**Authors:** Anli Liu, Siqi Li, Nana Cen, Fuying Mao, Ruixia Yang, Linfei Li, Hong Sui, Yunsheng Zhao

**Affiliations:** 1 School of Pharmacy, Ningxia Medical University, No. 1160, Shengli South Avenue, Xingqing District, Yinchuan, 750004 People’s Republic of China; 2 Ningxia Research Center of Modern Hui Medicine Engineering and Technology, Yinchuan, People’s Republic of China; 3 Key Laboratory of Hui Ethnic Medicine Modernization, Ministry of Education, Yinchuan, People’s Republic of China; 4 Wenxi County Meteorological Administration of Shanxi Province, Yuncheng, People’s Republic of China

**Keywords:** Plant domestication, Plant ecology, Environmental impact

## Abstract

Mineral nutrients play important roles in the growth and metabolism of *Ephedra intermedia*, and are affected by soil factors. Fifteen elements were measured from wild *E. intermedia* as well as their growing soils using inductively coupled plasma mass spectroscopy to investigate the influences and characteristics of herb elements. The pH, cation exchange capacity, humus and soil mechanical composition were also determined in rhizosphere soils. Results showed that *E. intermedia* stems contained high N, low P concentrations in macronutrients and high Fe in micronutrients, and enriched N, S, Cl, P and Sr from soils. The 15 herb elements were affected by one or more soil factors, and K, P, Zn, Fe and Mn were important soil elements that influenced the mineral accumulation of *E. intermedia*. This study was useful for the artificial cultivation of wild *E. intermedia*.

## Introduction

Ephedra herb (Ma-huang) is one of the traditional Chinese medicine (TCM) that has been applied to medical treatment for thousands of years in China^1^. The Chinese Pharmacopoeia (2020 Edition) stated that the dried herbaceous stem of *Ephedra intermedia* Schrenk et C.A Mey., *Ephedra sinica* Stapf and *Ephedra equisetina* Bge served as the traditional origin of Ma-huang^2^. Ma-huang is a stimulant, diaphoretic and antipyretic. Moreover, it is a highly popular, effective treatment for asthma and cough^3^. *E. intermedia* and *E. sinica* are the main resources of commercial Ma-huang. At present, *E. intermedia* is wild in China, mainly distributed in the drier regions of northwest China (such as Gansu, Ningxia)^4^ and an important plant to keep the desert grassland ecological balance. However, wild *E. intermedia* resources declined sharply because of extensive excavation^5^, and some legislation has been enacted to regulate wild *E. intermedia* harvesting stringently in China.

Mineral nutrients are inorganic chemical elements acquired primarily from the soil. Many mineral nutrients are imperative for plant growth^6^. They play prominent roles as active, textural components of metalloproteins and enzymes in plants’ viable cells^7,8^, and participate in various processes of plant metabolism^9–11^. Many mineral nutrients are important active ingredients of medicinal plants that are responsible for the therapeutic properties of herbs^12,13^. Many bioactive compounds can also be altered by these mineral nutrients via the changes of secondary metabolism in medicinal plants^14–16^. The efficacy of TCM is related to not only the organic compositions but also the types of inorganic elements and their mass fraction^17,18^. Accumulation of minerals in plants may also influence human physiology through the food chain^19^.

Mineral nutrients, mainly originating in soil, are absorbed by plants from soil solution in some form of inorganic ions or acids^20^. Soil type and composition influence the physiological processes of mineral nutrient adsorption, distribution and accumulation of plants^20–23^. Numerous researchers have proven that cation exchange capacity (CEC), pH, organic matter and carbonate concentrations of soils could affect absorption and bioavailability of elements^24–26^.

Mineral nutrients are essential for the growth of *E. intermedia* and the formation of curative materials. Much emphasis had been laid on studying the elemental interactions between soils and plants, and the major factors that control the process^27,28^. In the fruits of *E. sinica,* the total amount of iron (Fe), copper (Cu), zinc (Zn), manganese (Mn) and selenium was 4.01 µg g^−1^, which was higher than that of raisin, dried jujube and apricor^29^. We previously established the inorganic elemental fingerprints of 38 samples from *E. sinica*, *E. intermedia* and *E. przewalskii* by measuring the concentrations of nitrogen (N), phosphorous (P), potassium (K), sulfur (S), calcium (Ca), magnesium (Mg), Fe, Mn, sodium (Na), chlorine (Cl), strontium (Sr), Cu, Zn, boron (B) and molybdenum (Mo). The results showed that different *Ephedra* samples could be distinguished by these inorganic elements^30^. We also investigated the effects of rhizosphere soil on these elements in *E. sinica*; the results indicated that one or several factors, including soil organic matter, silt, sand and pH, had a significant effect on K, N, Cl, Cu, Mn, Na, B, Sr and Mo concentrations in *E. sinica*^31^. The concentration of N, K, Ca, Sr, Mn, Zn and Cu in *E. sinica* was positively related to that in soil. However, few works reported on the relationships between *E. intermedia* and its growing soil. Effects of soil factors on mineral nutrients of *E. intermedia* are well indistinct. In the present paper, we (1) measured 15 mineral nutrient concentrations in the dried herbaceous stem of *E. intermedia* and its growing soil; (2) determined soil pH, CEC, humus, sand, silt and clay concentration; (3) investigated the characteristics of elements in *E. intermedia* and soil samples; (4) studied the relationship between plant elements and soil factors and (5) revealed the important soil factors influencing mineral accumulation in *E. intermedia*.

## Material and methods

### Material

**Figure 1:**
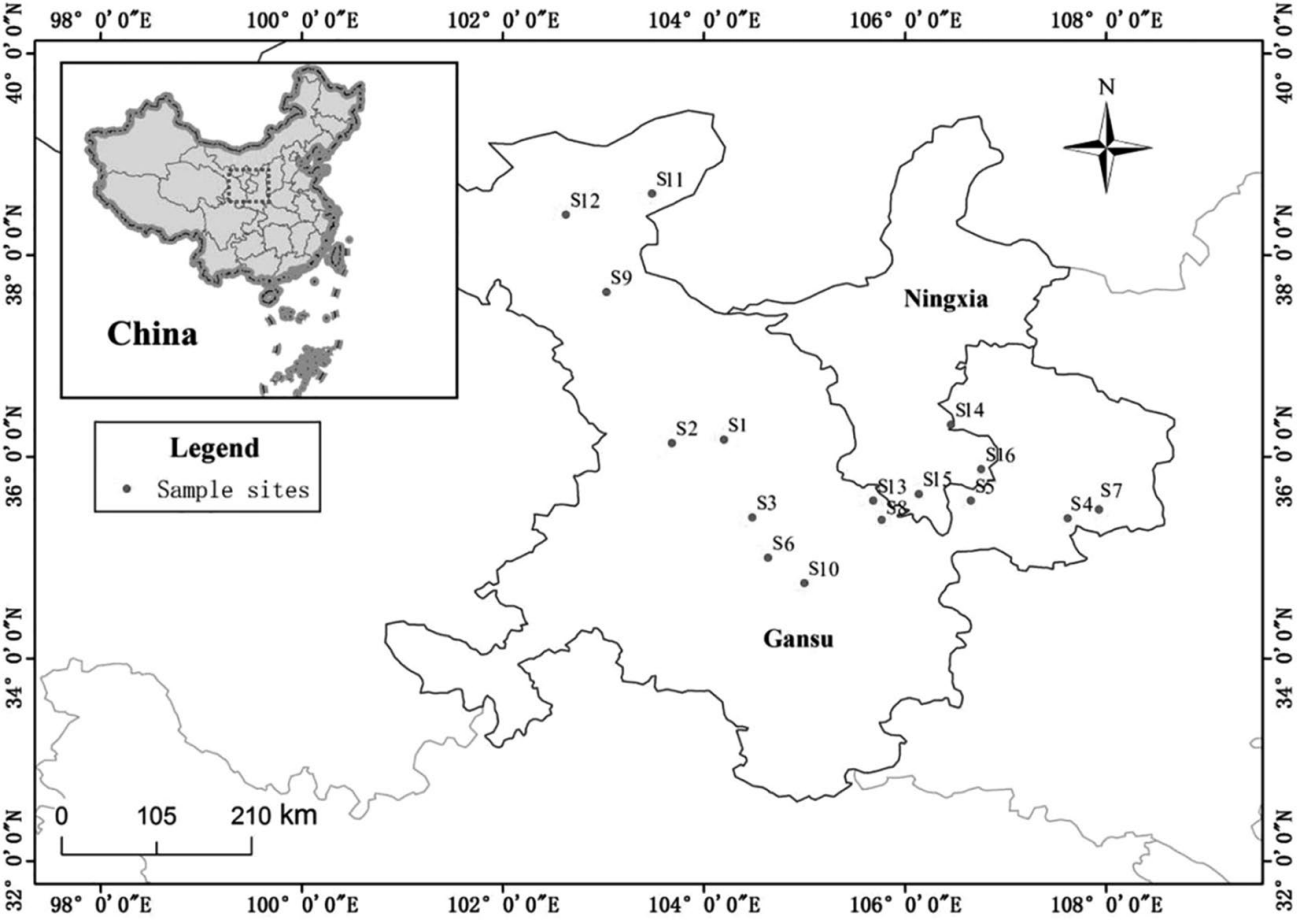
Growing locations of the *E. intermedia* samples involved in two provinces of China for this study. Maps generated by ArcGIS 10.0.

Samples of *E. intermedia* were collected from Ningxia and Gansu Province of China in 2012 (See Fig. 1 and Supplementary Table S1). The herb samples were taken from green stems of wild *E. intermedia.* All plant materials were identified as herbaceous stems of authentic *E. intermedia* by Professor Hong Sui. Five quadrats were selected for collection at each sampling point (100 g plant per quadrat) to ensure that all plant samples were representative. The sampling area was 1 m × 1 m for every quadrat, and the distance between two quadrats was over 200 m. All voucher specimens were deposited in Room 207, Ningxia Research Centre of Modern Hui Medicine Engineering and Technology (Yinchuan, Ningxia, China). Soil samples were collected at the root growing location of collected plants in each sampling point (500 g soil per quadrat), and surface mulch was removed. Next, 2,500 g of soil samples was obtained at a topsoil depth of 20 cm, mixed fully, packed and labeled in each sampling point.

Standard stock solutions as well as internal standard solution were purchased from Beijing General Research Institute for Nonferrous Metals in China for inductively coupled plasma mass spectroscopy (ICP-MS). HNO_3_, HF and H_2_O_2_ were of analytical reagent grade. Ultrapure water (Millipore Co., Ltd., U.S.) was used at a conductance of 18.25 MΩ cm. All glassware and plastic containers were thoroughly washed with HNO_3_ and cleaned with ultrapure water prior to use.

### Sample preparation and measurement

The samples were prepared using the following protocols. Herbaceous stems of *E. intermedia* were carefully rinsed with ultrapure water to remove dust particles. Soil samples were dried in an oven to reach a constant level. Dried samples were triturated into a homogeneous fine powder with a uniform particle size prior to analysis.

For microwave-assisted digestion technique, a programmable 1,200 W microwave (MARS 5, CEM Co., Ltd., U.S.A) served as the digestion system, and 5 ml of HNO_3_ along with 3 ml of H_2_O_2_ were mixed with 1.000 g of plant samples in Teflon digestion vessels. Then, the following procedure was performed in order: 20 min in 120 ℃, 20 min in 160 ℃ and 45 min in 180 ℃. For analysis of elements in soil, 0.5000 g of sample was placed in Teflon digestion vessels with a mixture of HNO_3_ (15 mL), HF (1 mL) and H_2_O_2_ (2 mL). The following procedure was performed in order: 25 min in 150 ℃, 30 min in 170 ℃ and 80 min in 200 ℃. After digestion, the solution was dried at 150 ℃. The dry digest was cooled to room temperature, attenuated with ultrapure water and transferred to volumetric flasks to a final volume of 10 mL. Blank samples were produced using a uniform analytical process without the plants or soils.

Concentrations of mineral factors in the plant and soil samples were measured by ICP-MS (NexION 300D, PerkinElmer Instrument Co., U.S.) using JIS K0133-2007 Method^30^. All determinations were conducted in triplicate.

### Soil characterisation measurement

All dried soil samples were ground and then sifted for analysis of physicochemical properties. Soil features studied included pH, CEC, humus and soil mechanical composition (SMC, involving silt, clay and sand). These characteristics were measured using the Chinese national standard methods in connection with soil^32–34^.

### Statistical analysis

The experimental data were processed using Excel 2010 and SPSS 21.0 statistical package. Multivariate data analysis techniques were applied, including correlation analysis (CA), coefficient of variation (CV), principal component analysis (PCA), hierarchical cluster analysis (HCA), multivariate analysis of variance (MAV) and discrimination analysis (DA).

CA and MAV were used to analyse the correlations and differences between the data by Pearson criteria and least significant difference (LSD), respectively. Statistical significance was recognised as a P value of less than 0.05, and high statistical significance had a *P* value of less than 0.01. CV was adopted to compute the deviation of element concentration levels between the soil and the plants^35^. PCA was conducted to extract factors from multivariate data, which made the data matrix less dimensional; original variables were converted into principal component data points^36^. HCA is used for grouping original variables and is an unsupervised pattern recognition technique^37^. HCA was performed using between-group linkage and squared Euclidean distance as the clustering method in this study. DA is a supervised pattern recognition technique to categorise samples by creating new variables^38^, thereby making the variance maximum between classifications and minimum within classifications.

## Results and discussion

### Soil characterisations

Soil characterisations including pH, CEC, SMC (silt, clay and sand) and humus from different samples were measured and listed in Supplementary Table S2. The soil samples were neutral or slightly alkaline with the pH ranging from 7.22 to 8.34. The pH was related to the presence of soil protons, which affected the ionic exchange of soil ingredients such as humic acids. The soil CEC varied from 12.05 to 44.06 mmol kg^−1^. The sample with the maximal pH had the highest CEC (44.06), whereas the lowest CEC (12.05) was established in the neutral soil with a pH of 7.39. The CEC was the supreme quantity of cations at a certain soil pH level^39^ and contributed to the soils’ potential information to adsorb or release cations^19^.

The SMC distribution (0.05 ≤ sand < 2, 0.002 ≤ silt < 0.05 and clay < 0.002 mm) varied significantly in the soils (6.42–24.06% clay, 0.64–9.96% silt and 67.34–90.3% sand). The average humus concentrations were 0.71% in the soil samples (in the range of 0.24–2.93%). They differed in various sampling areas. All of them were important for estimating the fitness of the soil and provided specific environment for plant growth.

### Element concentrations in *E. intermedia* and in the soil samples

Concentrations of 15 mineral nutrients were measured in *E. intermedia* and soil samples (Supplementary Tables S3 and S4) by ICP-MS. Elemental concentration variations are listed in Supplementary Table S5.

The average element concentrations in *E. intermedia* samples followed the order Mo < Cu < Zn < B < Mn < Na < Sr < Cl < Fe < P < Mg < S < Ca < K < N. The elements of P, K, N, Ca, S and Mg were the macronutrients in the plants, and concentrations of N and K were much higher than those of other macronutrients and more than 6,000 ppm. The elements of Fe, Cl, Sr, Na, Mn, B, Zn, Cu and Mo were the trace elements in the plants, and concentrations of Fe, Cl, Sr and Na were much higher than those of other trace elements and more than 133 ppm. Levels of mineral nutrients differed evidently according to plant sampling sites^40,41^. The average total concentration of elements was 45,148.58 ppm with a variation from 34,628.23 ppm to 55,123.86 ppm in 16 plant samples. The maximal concentration was obtained for N, which ranged from 18,863.61 ppm to 34,283.81 ppm and possessed 59.77% of the elemental total concentration in *E. intermedia*. The ascending order of element concentrations in *E. intermedia* was consistent with that in *E. sinica*; nevertheless, the order of S was after that of Ca in *E. sinica*^31^. The average total concentration of elements in *E. intermedia* was more than that in *E. sinica* (34,863.55 ppm), and the N ratio in the elemental total concentration was higher than that in *E. sinica* (55.98%)^31^. As an essential macronutrient, P showed a lower concentration (mean 843.20 ppm) compared with the other macronutrients (mean ≥ 2,258.57 ppm). Iron had the maximum concentration in micronutrients, which varied from 365.03 ppm to 1,497.56 ppm; its mean was 780.98 ppm in *E. intermedia*, which was higher by 337.61 ppm than that in *E. sinica*^31^. Iron concentration in *E. intermedia* samples exceeded the reference range (17–50 ppm) for general plants in agricultural lands recorded by Kabata–Pendias and Mukhrjee^42^, and particular mechanisms may exist for *E. intermedia* to obtain more Fe from its growing soil. Molybdenum concentration was less than that of other elements in *E. intermedia*, which was probably connected with its low availability and high affinity in soils.

The average element concentrations in the soil samples followed the order Mo < Cu < B < Cl < Zn < Sr < S < P < N < Mn < Mg < Fe < K < Na < Ca. This order was different from that in the plants. The average total concentration of elements was 92,635.34 ppm with the differentiation from 41,553.26 ppm to 132,697.48 ppm in the soils. The soil element of maximal concentration was Ca, its mean was 66,026.66 ppm, and it accounted for 71.28% of the total concentration of elements. The top six element concentrations in the soil were Mn, Mg, Fe, K, Na and Ca; those in *E. intermedia* were P, Mg, S, Ca, K and N. Concentrations of Ca, Mg and K were much high in the soils or *E. intermedia*. As the plant required macronutrients, the demands of N, P and S were large for *E. intermedia*. Supplementary Tables S3 and S4 show that the concentrations of N, P and S in *E. intermedia* were higher than those in the soils; thus, *E. intermedia* were able to transport these three elements actively to meet their own needs. Concentrations of Mo and Cu in *E. intermedia* and its growing soil were lower than those of other elements, and their sequences of elements were the same in *E. intermedia* and its growing soils. However, concentrations of Mo and Cu in the soil were higher than those in *E. intermedia*. Thus, the absorption of Mo and Cu in *E. intermedia* should not be the result of passive transport.

### Element variation in *E. intermedia* and its growing soil samples

**Figure 2:**
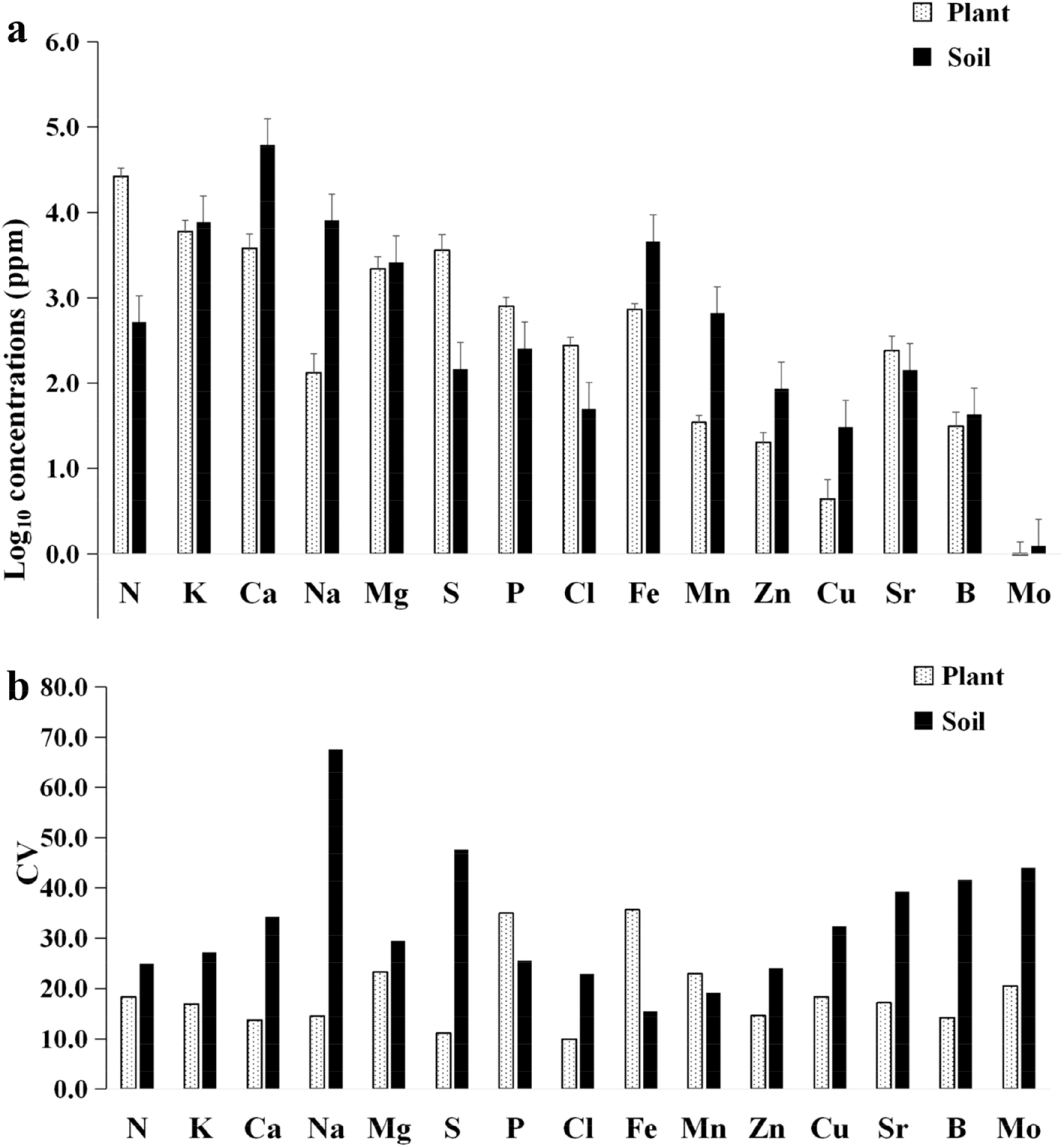
Elements mean concentrations and coefficients of variation of plants and soils. (**a**) Average concentration of 15 mineral elements in *E. intermedia* and soil samples. (**b**) Elements coefficients of variation in *E. intermedia* and soil samples.

For visual observation, the concentrations of plant and soil elements were converted logarithmically (Fig. 2a), and their CV (ratio of standard deviation to mean of all samples) was presented (Fig. 2b). Elemental concentrations varied greatly between the plants and the soils. Concentrations of Sr, Cl, P and S as well as N were significantly higher in *E. intermedia* (*P* < 0.01) than in the soils, and other elements in *E. intermedia* were significantly lower (*P* < 0.05) than those in the soil samples except for Mg. The concentration of Mg was not significantly different between the plant samples and the soil samples.

CVs in response to the plant samples decreased in the sequence Fe > P > Mg > Mn > Mo > N > Cu > Sr > K > Zn > Na > B > Ca > S > Cl. CVs of the soil samples followed the order Na > S > Mo > B > Sr > Ca > Cu > Mg > K > P > N > Zn > Cl > Mn > Fe. CVs in *E. intermedia* were evidently different from those in the soil samples. The top six elements of CVs in *E. intermedia* were Fe, P, Mg, Mn, Mo and N. The bottom six elements of CVs in the soil were Fe, Mn, Cl, Zn, N and P. Concentrations of Fe, P, Mn and N in *E. intermedia* were clearly unstable, and the CVs were large. By contrast, their concentrations in the soil were stable, and the CVs were small. Therefore, the absorption of these elements might be greatly affected by the aboveground environmental factors in the growth location of *E. intermedia*. The bottom six elements of CVs in *E. intermedia* were Cl, S, Ca, B, Na and Zn. The top six elements of CVs in the soil were Na, S, Mo, B, Sr and Ca. Thus, concentrations of S, Ca, B and Na were stable, and their CVs were small in *E. intermedia*. By contrast, their concentrations were not stable, and the CVs were large in the soil samples. Therefore, the absorption of S, Ca, B and Na might be mainly related to biological characteristics of *E. intermedia* rather than its soil environments. The largest CV and ratio of maximum to minimum (RMM) were observed in Fe of *E. intermedia* (Supplementary Table S5). Iron shows an important influence on animal energy metabolism, and it is the component of human hemoglobin and myoglobin^43^; Iron might be a curative indicator for *E. intermedia*. Phosphorous was similar to Fe in *E. intermedia*, that is, its RMM and CV were lower than those of Fe but higher than those of other elements. The maximum and minimum concentrations were higher for N, S, P, Cl and Sr in *E. intermedia* than in the soil samples. Potassium and magnesium minimum concentrations in herbs were also more than both in the soil samples. Overall, most elements’ maximum or minimum concentrations and CVs in the soils were higher than those in *E. intermedia*. CVs displayed a high element dependence for *E. intermedia* or soils. CVs of Fe and P varied by > 34% in *E. intermedia*, whereas CVs of Zn, Na, B, Ca, S and Cl varied by < 15%, which meant they were relatively constant. However, CVs of Na, S, Mo and B were > 41% in the soils, whereas CVs of Mn and Fe were < 20%. CV was consistent with the stability of limiting elements in *E. intermedia* or the soil sample. The CV of Na in the soils was approximately 70%, which was the largest among soil elements; thus, it was the most sensitive element in the soil. However, the CV of Na in *E. intermedia* was steady and only 14.15%.

### Element accumulation in *E. Intermedia*

Enrichment coefficients were computed to indicate the uptake and storage behaviour of elements in wild *E. intermedia*, which reflected the soil–plant system element migration rate to some extent, and investigate the capacity of *E. intermedia* to store elements from their growing soil. Enrichment coefficient (EC) was described in the following formula for the soil–plant system^27^:

$$ {\text{EC}} = \left[ {{\text{M}}_{{{\text{plant}}}} } \right]/\left[ {{\text{M}}_{{{\text{soil}}}} } \right], $$

where [M_plant_] represents the average concentration of an element in the herbaceous stem of *E. intermedia*, and [M_soil_] represents the same element average concentration in the soils.

**Figure 3:**
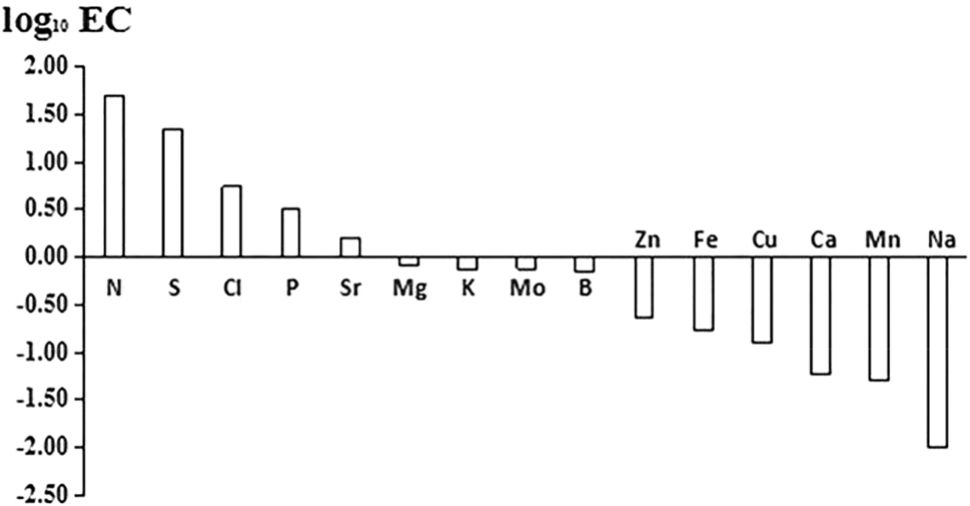
Enrichment coefficients of mineral elements from soils to plant.

ECs of mineral nutrients of *E. intermedia* varied widely (Fig. 3). Element ECs followed the order Na < Mn < Ca < Cu < Fe < Zn < B < K < Mo < Mg < Sr < P < Cl < S < N. ECs of N, S, Cl, P and Sr were greater than 1 and higher than those of other elements. Concentrations of these elements in *E. intermedia* were all higher than those in the soil, which was the result of the active transport of *E. intermedia*. ECs of Na, Mn and Ca were less than 0.06 and smaller than those of other elements. Concentrations of these elements in the soil were much higher than those in *E. intermedia*. The ascending order of ECs in *E. intermedia* was nearly consistent with that of *E. sinica* other than P, Cl, K, Mo, Ca and Fe^31^. *E. intermedia* accumulated the highest amount of N with an EC of 50.52 and showed a good tendency to accumulate S, Cl, P and Sr, whose ECs were 22.42, 5.45, 3.21 and 1.60 respectively, which might be involved in meeting the specific nutrition needs of *E. intermedia*. ECs of other elements were low (< 1), and Na accumulation was the lowest with a logarithm EC of − 2.

### Correlation analysis between plant elements and soil compositions

CA between *E. intermedia* and the soil samples was carried out to investigate the influence of the soil on the plant. The results are shown in Tables 1 and 2. Of the 225 element CAs between *E. intermedia* and the soil samples (Table 1), 49 had a significant correlation; P, K, Mn and Ca in *E. intermedia* were positively correlated with those in the soils. In *E. sinica,* K, Mn and Ca were also related to those in the soils, and the same CAs showed 102 significant correlations; thus, elements of *E. intermedia* were affected to a lesser extent by soil elements than those of *E. sinica*^31^*.* Chlorine and strontium in the plants were not related to any element in the soil samples. Nitrogen as well as Cu in *E. intermedia* were correlated with seven elements in the soils, and the correlative number of elements was maximal in the plants. Molybdenum, potassium and phosphorous in the herbs were related to six, five and five elements in the soils, respectively. All correlative elements in the plants were significantly positively related to the designated elements in the soils except S. Only S in the plants was significantly negatively correlated with Zn in the soils. Iron in the soil samples was associated with eight elements in *E. intermedia* and held the maximal number of correlative elements. Manganese and zinc in the soils were correlated with seven and six elements in the herbs, respectively. Sulfur and chlorine in the soils were not related to any element in the plants. Therefore, N, P, K, Cu and Mo in *E. intermedia* were easily influenced by elements in the soils, and Fe, Mn and Zn in the soils were important for *E. intermedia*.

**Table 1:** Correlation analysis between plant and soil elements.

Plant	Soil														
	Ca	Na	K	Fe	Mg	Mn	N	S	P	Sr	Zn	Cl	B	Cu	Mo
N	− 0.036	0.570*	0.762**	0.561*	0.641**	0.498*	0.307	0.155	0.596*	0.469	0.759**	0.013	0.453	0.461	− 0.203
K	− 0.059	0.536*	0.628**	0.398	0.692**	0.370	0.179	0.238	0.398	0.510*	0.617*	− 0.108	0.388	0.356	− 0.159
S	− 0.131	− 0.141	− 0.335	− 0.296	− 0.089	− 0.134	− 0.060	0.273	− 0.190	− 0.067	− 0.503*	− 0.449	− 0.487	− 0.453	− 0.187
Ca	0.571*	− 0.172	0.086	0.321	− 0.106	0.019	− 0.151	− 0.183	0.022	0.058	0.017	0.321	− 0.077	0.142	0.514*
Mg	0.284	0.052	0.186	0.501*	0.081	0.352	0.170	0.066	0.226	− 0.098	0.244	0.063	0.360	0.449	0.730**
P	− 0.241	0.653**	0.393	0.227	0.020	0.530*	0.442	0.012	0.696**	0.561*	0.432	− 0.204	0.912**	0.127	0.102
Fe	− 0.063	0.608*	0.251	− 0.16	0.176	0.172	0.054	0.268	0.400	0.940**	0.119	− 0.272	0.477	− 0.262	− 0.271
Cl	0.084	0.239	0.423	0.300	0.146	0.342	0.161	− 0.387	0.497	0.099	0.392	− 0.180	0.141	0.174	− 0.109
Sr	− 0.011	0.431	− 0.101	− 0.036	0.105	0.268	0.217	0.364	0.203	0.464	− 0.105	− 0.164	0.292	− 0.187	0.211
Na	− 0.119	0.180	0.309	0.522*	0.196	0.417	0.341	0.133	0.307	− 0.135	0.438	0.245	0.265	0.497	0.283
Mn	− 0.296	− 0.062	0.339	0.627**	0.251	0.834**	0.801**	0.057	0.414	− 0.319	0.409	− 0.051	0.124	0.430	0.135
B	− 0.093	0.269	0.455	0.630**	0.379	0.592*	0.486	0.245	0.431	0.049	0.605*	0.216	0.337	0.559*	0.111
Zn	− 0.377	0.305	0.443	0.568*	0.292	0.870**	0.829**	0.083	0.693**	0.111	0.432	− 0.095	0.491	0.359	0.115
Cu	− 0.335	0.440	0.621*	0.546*	0.492	0.765**	0.659**	0.032	0.657**	0.154	0.690**	− 0.059	0.525*	0.461	− 0.086
Mo	− 0.376	0.230	0.448	0.707**	0.349	0.792**	0.801**	0.076	0.562*	− 0.016	0.537*	0.185	0.465	0.555*	0.153

*Correlation is significant at the 0.05 level (2-tailed). ** Correlation is significant at the 0.01 level (2-tailed).

**Table 2:** Correlation analysis between plant elements and soil character factors. (CEC: cation exchange capacity).

Plant	Soil					
	Sand	Silt	Clay	pH	Humus	CEC
N	− 0.270	0.265	0.244	0.182	0.405	0.411
K	− 0.277	0.321	0.227	0.320	0.256	0.312
S	0.390	− 0.356	− 0.364	0.041	− 0.376	0.34
Ca	− 0.032	0.237	− 0.067	0.154	− 0.158	0.073
Mg	− 0.326	0.452	0.232	0.576*	0.354	0.273
P	−0.505*	0.435	0.484	0.347	0.259	0.561*
Fe	− 0.551*	0.405	0.561*	0.473	− 0.339	0.544*
Cl	0.005	0.345	− 0.167	− 0.026	0.214	0.151
Sr	− 0.648**	0.502*	0.648**	0.500*	− 0.279	0.543*
Na	− 0.031	− 0.106	0.092	0.201	0.495	0.194
Mn	0.050	0.097	− 0.113	− 0.192	0.838**	0.03
B	− 0.374	0.290	0.373	0.061	0.561*	0.099
Zn	− 0.270	0.251	0.250	0.165	0.709**	0.470
Cu	− 0.219	0.277	0.169	0.063	0.735**	0.246
Mo	− 0.314	0.257	0.307	− 0.066	0.805**	0.255

*Correlation is significant at the 0.05 level (2-tailed). **Correlation is significant at the 0.01 level (2-tailed).

The uptake of mineral nutrients in a plant was controlled by soil characterisations and plant biological properties^20^. CAs between herb elemental concentrations, and soil characterisation factors demonstrated that humus, CEC and sand were more important than other characterisation factors in the soils (Table 2). Soil humus was associated with Zn, Mn, Cu, B and Mo in *E. intermedia*, and had a maximal number of correlative elements in the plants. CEC in the soils was correlated with Sr, Fe and P in the herbs. All correlative parameters in the soils were significantly positively correlated with specific elements in the plants except sand, and sand in the soils was negatively proportional to Sr, Fe and P in the plants. Additionally, clay with Fe and Sr in herbs, pH with Mg and Sr, and silt with Sr were positively correlated. Strontium in *E. intermedia* was correlated with all characterisation factors in the soils except humus. Iron in the herbs was correlated with three soil characterisation factors, including CEC, sand and clay. Phosphorous in the herbs was related to sand and CEC in the soils.

The above CA indicated that soil elements and characterisation factors affected the absorption of specific elements in the plants at different levels. Element concentration in *E. intermedia* was correlated with several soil elements or characterisation factors. PCA was conducted to clarify the possible influence of soil on plants and select the characteristic factors in soils and *E. intermedia*.

### Principal component analysis of plant elements and soil compositions

PCA was performed, and the results are shown in Tables 3 and 4. According to Table 3, the first six principal components (PC1 ~ 6) explained 87.511% of the soil data, and contributed 33.639%, 17.337%, 14.445%, 9.576%, 6.943% and 5.571% to the total variance. Soil PCA proved that P, K, Zn, B, silt and Fe were dominantly incorporated in PC1. Humus was highly weighted in PC2. Nitrogen along with Mn contributed to the maximum loading for PC3. PC4 was formed by variable Mo, and all element weights were low in PC5 and PC6. Therefore, the factors of silt, humus, N, Zn, K, B, P, Fe, Mo and Mn were considered characteristic factors in the growing soil of wild *E. intermedia*, and their loading weights were all more than 0.7 in PC1 ~ 6. These factors might be recognised as the most potent markers to illustrate the influence of soil on plant.

**Table 3:** Results from principal component analysis of soil samples. (CEC: cation exchange capacity).

Item	Principal Component					
	1	2	3	4	5	6
Sand	− 0.683	0.401	0.241	− 0.074	0.508	− 0.058
Silt	0.731	− 0.298	− 0.246	0.077	− 0.308	− 0.305
Clay	0.589	− 0.406	− 0.213	0.065	− 0.548	0.221
PH	0.371	− 0.618	0.055	0.416	0.378	0.061
Humus	0.472	0.754	0.294	0.049	− 0.147	− 0.051
CEC	0.631	− 0.433	0.316	0.288	0.312	− 0.120
N	0.297	0.232	0.852	0.058	− 0.314	− 0.078
K	0.841	0.283	− 0.217	− 0.278	0.194	− 0.147
Ca	0.120	− 0.088	− 0.753	0.354	0.129	− 0.392
Na	0.640	− 0.379	0.151	− 0.241	0.361	0.380
Mg	0.477	0.269	0.028	− 0.404	0.367	− 0.091
S	− 0.222	− 0.248	0.629	0.196	0.158	0.235
P	0.845	− 0.092	0.250	− 0.138	− 0.003	− 0.111
Cl	− 0.022	0.496	− 0.336	0.143	− 0.123	0.668
Fe	0.701	0.576	− 0.054	0.318	0.087	− 0.100
Mn	0.512	0.162	0.767	0.135	− 0.167	− 0.157
Zn	0.811	0.424	− 0.180	− 0.180	0.167	0.145
Cu	0.624	0.645	− 0.324	0.173	0.064	0.090
Sr	0.557	− 0.649	− 0.124	− 0.336	0.021	0.045
B	0.794	− 0.182	0.007	0.091	0.004	0.294
Mo	0.043	0.063	− 0.019	0.964	0.131	0.072
Variance (%)	33.639	17.337	14.445	9.576	6.943	5.571
Cumulative variance (%)	33.639	50.976	65.420	74.997	81.939	87.511

**Table 4:** Results from principal component analysis of *E. intermedia* samples.

Item	Principal component				
	1	2	3	4	5
N	0.798	0.280	− 0.202	0.132	0.442
K	0.711	0.420	− 0.024	0.141	0.467
Ca	− 0.042	− 0.141	0.719	− 0.213	0.348
Na	0.658	− 0.229	− 0.262	− 0.515	0.203
Mg	0.508	− 0.217	0.593	− 0.413	− 0.055
S	− 0.480	0.058	− 0.500	0.053	0.063
P	0.649	0.364	0.129	0.223	− 0.484
Cl	0.224	− 0.247	0.440	0.782	0.133
Fe	0.294	0.898	0.031	0.111	− 0.010
Mn	0.719	− 0.571	− 0.051	0.217	− 0.058
Zn	0.836	− 0.206	− 0.139	0.005	− 0.288
Cu	0.925	− 0.112	− 0.045	0.296	− 0.013
Sr	0.324	0.638	0.310	− 0.292	− 0.285
B	0.843	0.002	− 0.146	− 0.309	0.179
Mo	0.834	− 0.247	− 0.160	− 0.087	− 0.268
Variance (%)	41.375	14.855	10.712	10.126	7.374
Cumulative variance (%)	41.375	56.229	66.942	77.068	84.442

PCA results of *E. intermedia* showed that 15 original variables were converted to five PCs that totally contributed 84.442% of all element data sets (Table 4). The highly weighted factors under these PCs were Cu, B, Zn, Mo, N, Mn, K, Fe, Ca and Cl. PC1 explained 41.375% of the data variation and was mainly affected by Cu, B, Zn, Mo, N and Mn as the highly weighted factors. PC2, which contributed to 14.855% of the studied variances, displayed the main loading factor for Fe. PC3 and PC4 accounted for 10.712% and 10.126% of the total variance, respectively. Calcium was the major loading factor in PC3, and Cl was in PC4. Moreover, PC5 revealed 7.374% of the total variance, and all element weights were low in PC5. Thus, N, K, Mn, Zn, Cu, B, Ca, Mo, Fe and Cl were considered the characteristic minerals in *E. intermedia*, and their loading weights were all more than 0.7 in PC1 ~ 5.

### Hierarchical cluster analysis of plant and its growing soil samples

**Figure 4:**
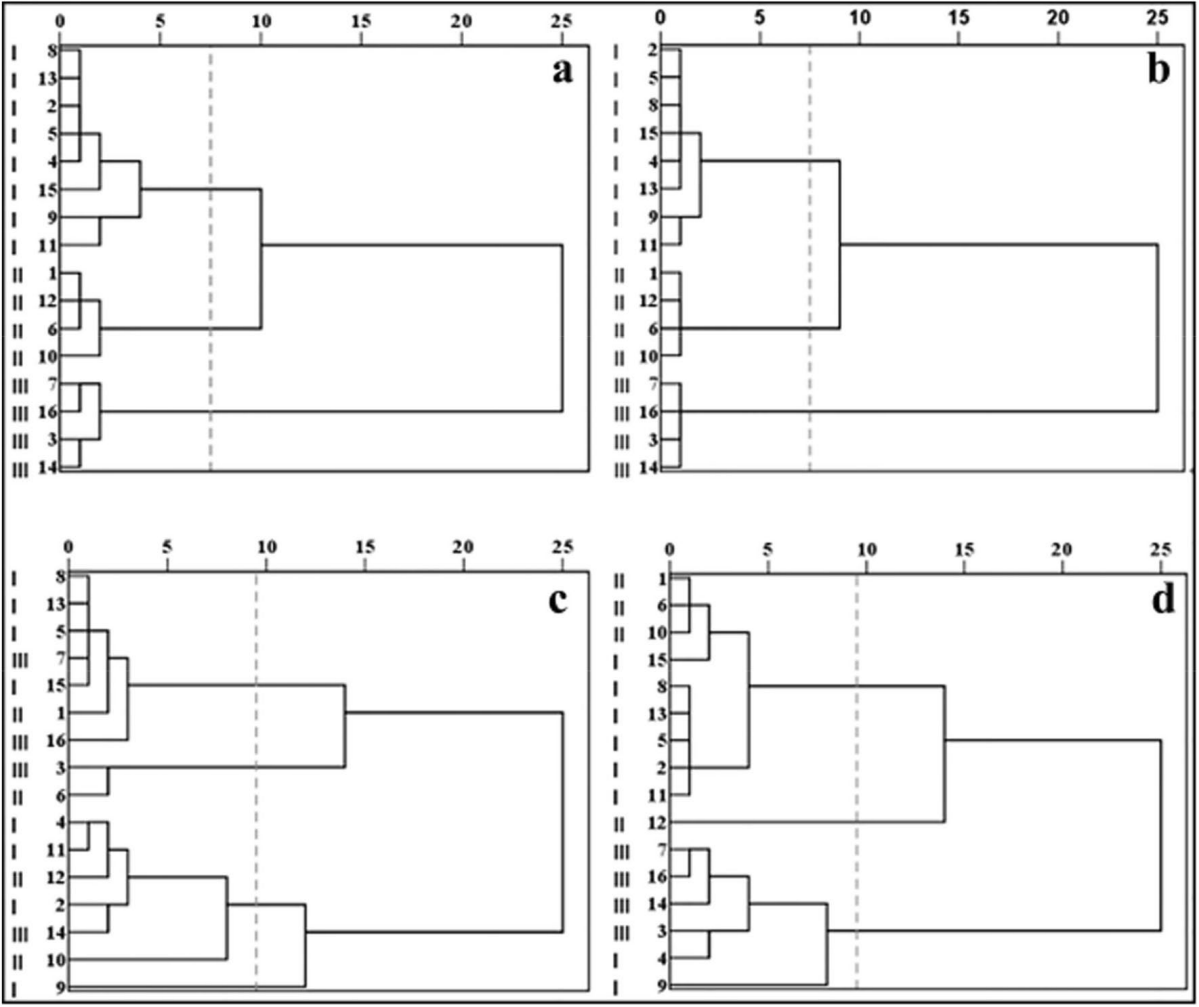
Dendrograms of hierarchical cluster analysis for *E. intermedia* and soil samples. (**a**) Dendrogram of herb samples based on 15 elements concentrations. (**b**) Dendrogram of herb samples based on 10 characteristic elements concentrations. (**c**) Dendrogram of soil samples based on 21 soil factors. (**d**) Dendrogram of soil samples based on 10 characteristic factors.

HCA was conducted for *E. intermedia* and soil samples based on the between-group linkage method. The results are shown in Fig. 4. According to the concentrations of 15 elements, 16 herb samples were clustered into three at a distance of 7.5 (Fig. 4a), which showed no difference from the cluster result according to 10 characteristic element concentrations from PCA (Fig. 4b). Based on 21 soil factors, 16 soil samples were grouped into four at a distance of 9.5 (Fig. 4c), which was not consistent with those of *E. intermedia* samples. However, based on the 10 soil characteristic factors from PCA, 16 soil samples were clustered into three at a distance of 9.5 (Fig. 4d), which approximately agreed with the clustered result of *E. intermedia* samples.

Sixteen soil samples were grouped into three for *E. intermedia* samples in HCA to reveal the effect of soil factors on herb mineral nutrients. Then, MAV was performed in three groups of soil samples. The difference was not significant (*P* > 0.05) among three soil groups according to 21 soil factors for analysis. However, based on 10 characteristic factors from the soil PCA, the three groups had a significant difference (*P* < 0.05). Thus, multiple comparisons of LSD were carried out. The results indicated that concentrations of K, P, Zn, Fe and Mn had a significant difference in the three groups of soil samples. The five elements of K, P, Zn, Fe and Mn in the soil samples were related significantly to 11 elements in *E. intermedia* samples, namely, N, K, S, Mg, P, Na, Mn, B, Zn, Cu and Mo, which all increased with the increase of soil K, P, Zn, Fe and Mn concentration except for S in *E. intermedia*. Thus, these soil elements might be the important factors that influenced the element concentrations in *E. intermedia*.

### Discrimination analysis of plant samples based on soil elements

**Figure 5:**
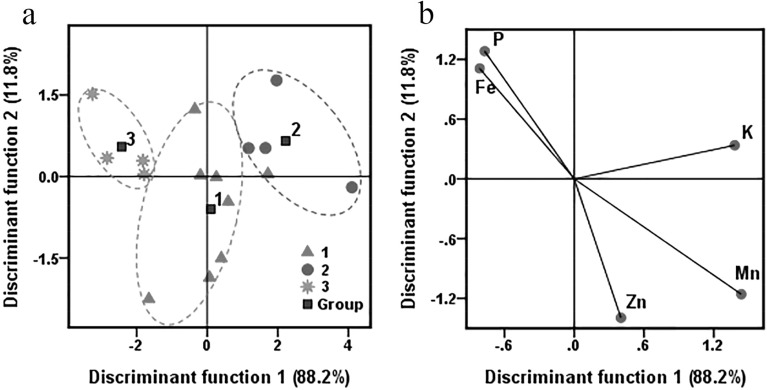
Discrimination analysis for 16 wild *E. intermedia* samples from different origins. (**a**) Scatter diagram of all wild *E.intermedia* samples based on five characteristic elements of soil. (**b**) Correlation chart between the selected soil variables and the discriminant functions.

The samples of *E. intermedia* were classified into three based on the HCA, and their growing soil samples were divided into the same three groups artificially. In the three soil groups, Zn, K, P, Fe and Mn showed significant differences. Therefore, differences of mineral nutrients and classification of *E. intermedia* samples were probably related to differences of Zn, K, P, Fe and Mn in the soil because these soil elements could affect the elemental absorption in *E. intermedia*. Discriminant analysis (DA) was conducted according to concentrations of Zn, K, P, Fe and Mn in the soils to test this relationship. The results are shown in Fig. 5 and Table 5. Distribution patterns of *E. intermedia* samples are displayed in the plot (Fig. 5a). The samples of *E. intermedia* from 16 origins were grouped into three in different spaces with a precision rate of 93.8%, which was consistent with the HCA results. Figure 5b shows the correlation chart of loading for the five variables (Zn, K, P, Fe and Mn) from the soil samples. Discriminant function 1 revealed 88.2% of the variance, contributing to primary separation for *E. intermedia* samples and having a high positive correlation with soil K and Mn. Discriminant function 2 (representing 11.8% of the variance) was positively related to soil Fe and P. According to the correlation between Fig. 4a and b, K, P, Fe and Mn were the most useful soil elements influencing *E. intermedia* samples.

**Table 5:** Grouped results of *E.intermedia* samples using discriminant analysis.

		Predicted groups from 5 variables of the soil samples				
		1	2	3	Total	Correct (%)
Assigned groups from *E. intermedia*	1	7	1	0	8	87.5
	2	0	4	0	4	100.0
	3	0	0	4	4	100.0
Total correct (%)						93.8

A cross-validation procedure was performed to investigate the classification or probability of *E. intermedia* samples (Table 5) and examine the dependability of DA. The results demonstrated that 87.5% of *E. intermedia* samples from Group 1 and 100% of *E. intermedia* samples from Groups 2 and 3 were appropriately clustered. These results were consistent with those obtained in the above research. Thus, soil elements of Zn, K, P, Fe and Mn were very important factors for the mineral accumulation in *E. intermedia*.

## Conclusions

*E. intermedia* was able to store nitrogen, phosphorous, sulfur, chlorine and strontium from its growing soils. Nitrogen, phosphorous, potassium, copper and molybdenum in *E. intermedia* were influenced to a greater extent by soil elements than other elements. Strontium in *E. intermedia* was mainly influenced by soil characterisation factors. Phosphorous, potassium, iron, zinc and manganese in the soil samples were related significantly to 11 elements in *E. intermedia* samples, namely, N, P, K, S, Mg, Mn, Cu, Mo, Na, Zn and B, and were considered markers to illustrate the influence of soil on *E. intermedia*. This study contributed to the mineral supervision of *E. intermedia* by regulating soil factor levels.

## Supplementary information

Supplementary file 1.

## Data Availability

Datasets generated during and/or analysed during the current study are available from the corresponding author on reasonable request.
