# Supplementary material for: Concentration of fifteen elements in herbaceous stems of Ephedra intermedia and influence of its growing soil
Source: Sci Rep. 2020 Sep 15;10:15077. doi: 10.1038/s41598-020-72048-w (PMC7494874; doi:10.1038/s41598-020-72048-w)
Supplement: Supplementary file 1 [file 41598_2020_72048_MOESM1_ESM.doc]

**Concentration of fifteen elements in herbaceous stems of *Ephedra intermedia* and influence of its growing soil**

Anli Liu, Siqi Li, Nana Cen, Fuying Mao, Ruixia Yang, Linfei Li, Hong Sui, Yunsheng Zhao^*^

**Supplementary Material**

Table S1. Samples sources of *E. intermedia* and their growing soils in this work

| Samples | Province | Locations | Longitude | Latitude | Altitude (m) | Parent material | Soil type |
| --- | --- | --- | --- | --- | --- | --- | --- |
| 1 | Gansu | Anning | E 103°72' | N 36°10' | 1540 | A[alluvial](../../../../D:/Users/zys/AppData/Local/Youdao/Dict/8.9.3.0/resultui/html/index.html#/javascript:;) material | White loessial soil |
| 2 | Rensishan | E 103°40'58.32" | N 36°08'00.58" | 1620 | A[alluvial](../../../../D:/Users/zys/AppData/Local/Youdao/Dict/8.9.3.0/resultui/html/index.html#/javascript:;) material | White loessial soil |
| 3 | Huining | E 104°29' | N 35°24' | 1944 | Secondary loess | Irrigated loessial soil |
| 4 | Jingchuan | E 107°37'04.29" | N 35°23'29.86" | 1094 | Malan loess | Soft loessial soils |
| 5 | Kongtong | E 106°39'24.15" | N 35°33'53.99" | 1462 | Malan loess | Soft loessial soils |
| 6 | Longxi | E 104°38'21.01" | N 35°00'00.07" | 1760 | Loess | Flaying kastanzens |
| 7 | Ningxian | E 107°54′05.86″ | N 35°31′25.09″ | 1034 | Lishi loess | White greyzem |
| 8 | Qinan | E 105°62'37.60" | N 34°98'57.06" | 1600 | Secondary loess | Irrigated loessial soil |
| 9 | Tianzhu | E 103°01'51.08" | N 36°97'67.75" | 2800 | Loess-like material | Tianzhu dark chestnut soil |
| 10 | Wushan | E 104°59'56.78" | N 34°44'52.00" | 1584 | Loess | Ma-cinnamon loess |
| 11 | Liangzhouqu | E 102°88'69.63" | N 37°96'42.30" | 1450 | Loess | Light chestnut soil |
| 12 | Yongchang | E 101°97'46.09" | N 38°24'10.82" | 2913 | Loess-like material | Tianzhu black soil |
| 13 | Zhuanglang | E 106°03′62.63″ | N 35°41′39.56″ | 1850 | Malan loess | Haplic kastanzens |
| 14 | Ningxia | Guyuan | E 106°27'36.73" | N 36°19'06.57" | 1877 | Malan loess | Light loessial soils |
| 15 | Longde | E 106°08'12.63" | N 35°37'57.64" | 2261 | Mudstone or shale weathering residual slope deposit | Thick dark kastanzen |
| 16 | Pengyang | E 106°49'59.38" | N 35°52'07.59" | 1486 | Malan loess | Mengyuan dark loessial soils |

Table S2. The characterisations of soil samples. (CEC: cation exchange capacity)

| Samples | Sand (%) | Silt (%) | Clay (%) | pH | Humus (%) | CEC |
| --- | --- | --- | --- | --- | --- | --- |
|  | 2-0.05 mm | 0.05-0.002 mm | <0.002 mm |  |  | (mmol kg^-1^) |
| Soil 1 | 90.30 | 3.28 | 6.42 | 7.67 | 0.62 | 31.73 |
| Soil 2 | 88.90 | 3.16 | 7.94 | 7.69 | 0.69 | 13.51 |
| Soil 3 | 87.74 | 3.84 | 8.42 | 7.82 | 0.32 | 28.37 |
| Soil 4 | 84.78 | 0.64 | 14.58 | 7.81 | 0.65 | 19.32 |
| Soil 5 | 74.42 | 7.00 | 18.58 | 7.67 | 0.61 | 21.61 |
| Soil 6 | 83.58 | 5.40 | 11.02 | 8.01 | 0.48 | 21.61 |
| Soil 7 | 79.94 | 5.12 | 14.94 | 7.39 | 0.35 | 12.05 |
| Soil 8 | 77.70 | 6.24 | 16.06 | 7.22 | 0.56 | 16.59 |
| Soil 9 | 90.22 | 2.36 | 7.42 | 7.56 | 0.53 | 24.53 |
| Soil 10 | 67.34 | 8.60 | 24.06 | 8.34 | 0.44 | 44.06 |
| Soil 11 | 83.58 | 5.48 | 10.94 | 7.61 | 0.26 | 18.54 |
| Soil 12 | 86.62 | 3.60 | 9.78 | 7.23 | 2.93 | 18.49 |
| Soil 13 | 74.26 | 5.20 | 20.54 | 7.42 | 0.65 | 20.24 |
| Soil 14 | 86.70 | 1.04 | 12.26 | 7.65 | 0.24 | 16.04 |
| Soil 15 | 69.06 | 9.96 | 20.98 | 8.10 | 1.57 | 29.57 |
| Soil 16 | 83.98 | 5.20 | 10.82 | 7.80 | 0.52 | 14.95 |

Table S3. Elemental concentrations (ppm) in *E. intermedia* from different regions

|  | N | K | Ca | Na | Mg | S | P | Cl | Fe | Mn | Zn | Cu | Sr | B | Mo | SUM |
| --- | --- | --- | --- | --- | --- | --- | --- | --- | --- | --- | --- | --- | --- | --- | --- | --- |
| LOD | 0.01 | 0.001 | 0.004 | 0.005 | 0.004 | 0.01 | 0.002 | 0.01 | 0.002 | 0.002 | 0.002 | 0.001 | 0.002 | 0.006 | 0.003 |
| plant 1 | 32504.09 | 7153.74 | 3754.43 | 129.39 | 1976.08 | 4021.69 | 598.26 | 296.93 | 710.55 | 35.12 | 19.33 | 4.73 | 245.37 | 31.38 | 0.93 | 51482.02 |
| plant 2 | 27957.07 | 6797.40 | 3267.25 | 128.31 | 2214.83 | 3061.43 | 985.15 | 319.44 | 665.46 | 35.14 | 17.18 | 5.20 | 196.22 | 30.12 | 0.75 | 45680.95 |
| plant3 | 18863.61 | 4284.02 | 4816.93 | 127.57 | 2830.34 | 3821.97 | 782.21 | 291.91 | 365.03 | 34.58 | 18.54 | 3.37 | 229.80 | 26.48 | 0.87 | 36497.23 |
| plant 4 | 29258.53 | 6709.87 | 3537.13 | 169.32 | 2526.97 | 3589.59 | 710.13 | 208.19 | 784.63 | 33.65 | 20.01 | 4.34 | 288.51 | 39.07 | 1.10 | 47881.04 |
| plant 5 | 27821.80 | 6065.96 | 3600.27 | 145.23 | 2232.74 | 3994.12 | 483.79 | 289.92 | 639.71 | 38.50 | 20.23 | 4.37 | 252.79 | 35.23 | 0.96 | 45625.62 |
| plant 6 | 32809.85 | 7125.22 | 4830.98 | 155.35 | 2663.94 | 3577.02 | 694.03 | 274.45 | 1033.12 | 33.06 | 20.18 | 4.47 | 215.67 | 32.99 | 0.82 | 53471.15 |
| plant 7 | 20996.08 | 4386.24 | 4502.89 | 122.72 | 2204.64 | 3122.18 | 582.59 | 282.65 | 551.92 | 29.44 | 18.14 | 3.81 | 243.41 | 29.77 | 0.92 | 37077.40 |
| plant 8 | 28136.77 | 6139.16 | 3547.10 | 127.74 | 1818.83 | 3311.91 | 1050.94 | 252.03 | 887.47 | 31.82 | 18.66 | 4.40 | 261.97 | 34.98 | 1.00 | 45624.78 |
| plant 9 | 23995.40 | 5546.68 | 3489.54 | 121.08 | 1923.99 | 4050.17 | 924.38 | 295.98 | 839.44 | 45.62 | 22.65 | 4.92 | 263.04 | 29.37 | 1.00 | 41553.26 |
| plant10 | 34283.81 | 7067.77 | 3698.63 | 147.45 | 2425.10 | 3623.73 | 1679.18 | 284.87 | 1497.56 | 31.99 | 25.00 | 5.31 | 317.30 | 34.95 | 1.21 | 55123.86 |
| plant 11 | 25912.70 | 6722.28 | 4445.75 | 116.89 | 1910.69 | 3944.90 | 684.71 | 291.78 | 976.84 | 31.24 | 18.99 | 4.00 | 253.59 | 28.35 | 0.96 | 45343.67 |
| plant 12 | 32868.37 | 6250.37 | 3660.15 | 169.80 | 2369.99 | 3424.01 | 1013.41 | 295.25 | 376.53 | 56.92 | 27.44 | 6.15 | 161.43 | 38.18 | 1.54 | 50719.54 |
| plant 13 | 28605.13 | 6348.01 | 3753.42 | 105.82 | 1635.11 | 3469.54 | 921.50 | 288.15 | 1056.13 | 33.01 | 18.35 | 4.34 | 248.67 | 29.67 | 0.94 | 46517.79 |
| Plant 14 | 19221.48 | 4473.09 | 3241.37 | 134.48 | 1690.22 | 4011.42 | 664.70 | 233.42 | 688.83 | 22.47 | 19.08 | 3.42 | 200.96 | 22.53 | 0.76 | 34628.23 |
| Plant 15 | 27189.28 | 7041.95 | 3940.41 | 137.59 | 3747.89 | 2853.22 | 1125.83 | 288.86 | 788.06 | 43.99 | 23.53 | 5.61 | 315.18 | 35.37 | 1.20 | 47537.97 |
| Plant 16 | 21329.20 | 4957.04 | 3276.22 | 104.82 | 1965.80 | 4210.67 | 590.33 | 257.11 | 634.32 | 27.30 | 16.63 | 3.27 | 211.89 | 27.42 | 0.80 | 37612.82 |

Table S4. Elemental concentrations (ppm) in rhizosphere soil samples from different regions

|  | N | K | Ca | Na | Mg | S | P | Cl | Fe | Mn | Zn | Cu | Sr | B | Mo | SUM |
| --- | --- | --- | --- | --- | --- | --- | --- | --- | --- | --- | --- | --- | --- | --- | --- | --- |
| LOD | 0.01 | 0.001 | 0.004 | 0.005 | 0.004 | 0.01 | 0.002 | 0.01 | 0.002 | 0.002 | 0.002 | 0.001 | 0.002 | 0.006 | 0.003 |
| soil 1 | 486.47 | 9979.07 | 77219.55 | 16254.00 | 4704.80 | 177.81 | 266.42 | 47.80 | 5031.14 | 646.71 | 100.91 | 35.77 | 145.19 | 31.49 | 0.97 | 115128.10 |
| soil 2 | 389.02 | 8583.09 | 53776.84 | 15176.25 | 2371.01 | 111.54 | 264.18 | 51.56 | 4136.00 | 574.69 | 110.92 | 35.78 | 135.98 | 57.95 | 0.99 | 85775.80 |
| soil 3 | 455.61 | 5791.31 | 101126.08 | 3949.00 | 1100.26 | 103.62 | 224.40 | 51.66 | 5163.48 | 620.36 | 72.00 | 35.21 | 74.59 | 46.01 | 3.02 | 118816.61 |
| soil 4 | 572.00 | 4849.53 | 42378.00 | 8982.65 | 2566.86 | 348.64 | 160.33 | 68.89 | 4571.00 | 669.45 | 77.79 | 34.27 | 92.58 | 37.38 | 2.01 | 65411.38 |
| soil 5 | 501.18 | 8541.56 | 81627.03 | 7412.25 | 2612.68 | 90.86 | 299.03 | 42.48 | 4996.64 | 676.71 | 93.35 | 39.21 | 130.71 | 25.46 | 0.88 | 107090.03 |
| soil 6 | 427.58 | 10072.94 | 107388.03 | 5525.50 | 2879.01 | 191.51 | 273.26 | 49.53 | 4832.56 | 675.15 | 95.36 | 33.67 | 211.10 | 40.83 | 1.45 | 132697.48 |
| soil 7 | 482.58 | 6446.00 | 74516.43 | 7526.00 | 2166.93 | 102.03 | 252.69 | 78.49 | 4218.06 | 606.96 | 81.22 | 29.98 | 112.78 | 30.66 | 1.20 | 96652.01 |
| soil 8 | 519.39 | 8638.26 | 78488.05 | 7386.50 | 2574.10 | 98.76 | 263.02 | 44.90 | 4474.39 | 624.38 | 93.84 | 32.40 | 181.34 | 55.31 | 0.99 | 103475.63 |
| soil 9 | 839.44 | 4050.17 | 23995.40 | 5546.68 | 1923.99 | 295.98 | 263.04 | 29.37 | 3489.54 | 924.38 | 45.62 | 4.92 | 121.08 | 22.65 | 1.00 | 41553.26 |
| soil 10 | 629.08 | 10525.46 | 53776.84 | 28989.50 | 2684.06 | 184.27 | 451.06 | 43.95 | 4895.66 | 836.55 | 113.33 | 32.58 | 306.00 | 98.78 | 1.32 | 103568.44 |
| soil 11 | 502.77 | 7955.45 | 46311.56 | 9625.25 | 3226.24 | 145.19 | 240.67 | 52.81 | 4540.59 | 635.42 | 85.28 | 31.82 | 199.60 | 36.04 | 1.04 | 73589.73 |
| soil 12 | 804.33 | 11691.37 | 39116.69 | 6865.50 | 3395.32 | 107.94 | 349.62 | 63.19 | 6337.85 | 918.09 | 131.29 | 57.96 | 76.94 | 59.61 | 1.29 | 69976.99 |
| soil 13 | 493.01 | 8782.05 | 79461.77 | 6837.00 | 2723.82 | 144.74 | 217.89 | 58.62 | 4687.35 | 614.03 | 92.56 | 33.62 | 215.80 | 48.50 | 0.89 | 104411.65 |
| soil 14 | 398.42 | 5901.18 | 53794.57 | 5776.25 | 2352.23 | 85.68 | 182.76 | 45.90 | 3413.72 | 447.54 | 59.33 | 22.88 | 140.75 | 39.10 | 0.92 | 72661.23 |
| soil 15 | 624.87 | 9302.45 | 76183.79 | 8775.25 | 3655.30 | 156.83 | 283.30 | 45.31 | 5281.32 | 787.13 | 97.32 | 44.80 | 161.03 | 66.23 | 2.04 | 105466.97 |
| soil 16 | 420.09 | 6548.27 | 67266.00 | 3950.25 | 2377.52 | 245.61 | 215.83 | 42.69 | 4004.35 | 556.48 | 69.86 | 27.80 | 129.61 | 34.73 | 1.07 | 85890.16 |

Table S5. Element concentrations (ppm) variations in *E.intermedia* and soil samples. (min: minimum; max: maximum; CV: coefficient of variation)

|  | *E.intermedia* samples | | | | | Soils samples | | | | |
| --- | --- | --- | --- | --- | --- | --- | --- | --- | --- | --- |
|  | Min | Max | Mean | Max/Min | CV | Min | Max | Mean | Max/Min | CV |
| N | 18863.61 | 34283.81 | 26984.57 | 1.82 | 18.34 | 389.02 | 839.44 | 534.12 | 2.16 | 24.83 |
| K | 4284.02 | 7153.74 | 6066.80 | 1.67 | 16.91 | 4050.17 | 11691.37 | 7978.64 | 2.89 | 27.25 |
| S | 2853.22 | 4210.67 | 3630.47 | 1.48 | 11.11 | 85.68 | 348.64 | 161.94 | 4.07 | 47.66 |
| Ca | 3241.37 | 4830.98 | 3835.15 | 1.49 | 13.77 | 23995.40 | 107388.03 | 66026.66 | 4.48 | 34.34 |
| Mg | 1635.11 | 3747.89 | 2258.57 | 2.29 | 23.23 | 1100.26 | 4704.8 | 2707.13 | 4.28 | 29.47 |
| P | 483.79 | 1679.18 | 843.20 | 3.47 | 34.99 | 160.33 | 451.06 | 262.97 | 2.81 | 25.61 |
| Fe | 365.03 | 1497.56 | 780.98 | 4.10 | 35.66 | 3413.72 | 6337.85 | 4629.60 | 1.86 | 15.43 |
| Cl | 208.19 | 319.44 | 278.18 | 1.53 | 9.95 | 29.37 | 78.49 | 51.07 | 2.67 | 22.91 |
| Sr | 161.43 | 317.30 | 244.11 | 1.97 | 17.15 | 74.59 | 306.00 | 152.19 | 4.10 | 39.25 |
| Na | 104.82 | 169.80 | 133.97 | 1.62 | 14.51 | 3949 | 28989.50 | 9286.11 | 7.34 | 67.62 |
| Mn | 22.47 | 56.92 | 35.24 | 2.53 | 22.89 | 447.54 | 924.38 | 675.88 | 2.07 | 19.18 |
| B | 22.53 | 39.07 | 31.62 | 1.73 | 14.19 | 22.65 | 98.78 | 45.67 | 4.36 | 41.55 |
| Zn | 16.63 | 27.44 | 20.25 | 1.65 | 14.58 | 45.62 | 131.29 | 88.75 | 2.88 | 23.96 |
| Cu | 3.27 | 6.15 | 4.48 | 1.88 | 18.30 | 4.92 | 57.96 | 33.29 | 11.78 | 32.45 |
| Mo | 0.75 | 1.54 | 0.99 | 2.05 | 20.47 | 0.88 | 3.02 | 1.32 | 3.43 | 43.99 |
| Total | 34628.23 | 55123.86 | 45148.58 |  |  | 41553.26 | 132697.48 | 92635.34 |  |  |
